# Evaluation and Validation of a Method for Determining Platelet Catecholamine in Patients with Obstructive Sleep Apnea and Arterial Hypertension

**DOI:** 10.1371/journal.pone.0098407

**Published:** 2014-06-09

**Authors:** Marcia C. Feres, Fatima D. Cintra, Camila F. Rizzi, Luciane Mello-Fujita, Altay A. Lino de Souza, Sergio Tufik, Dalva Poyares

**Affiliations:** 1 Psychobiology Department of Universidade Federal de São Paulo, Sao Paulo, SP, Brazil; 2 Cardiology Department of Universidade Federal de São Paulo, São Paulo, SP, Brazil; 3 Associação Fundo de Incentivo a Pesquisa – AFIP- São Paulo, São Paulo, SP, Brazil; University of Pécs Medical School, Hungary

## Abstract

**Background:**

Measurements of plasma and urinary catecholamine are susceptible to confounding factors that influence the results, complicating the interpretation of sympathetic nervous system (SNS) activity in the Obstructive sleep apnea (OSA) and arterial hypertension (HYP) conditions.

**Objective:**

In this study, we validated a test for platelet catecholamine and compared the catecholamine levels (adrenaline and noradrenaline) in urine, plasma and platelets in patients with OSA and HYP compared with controls.

**Methods:**

In the validation, 30 healthy, nonsmoking volunteers who were not currently undergoing treatment or medication were selected as the control group. One hundred fifty-four individuals (114 OSA, 40 non-OSA) were consecutively selected from the outpatient clinic of the Sleep Institute and underwent clinical, polysomnographic and laboratory evaluation, including the urinary, plasma and platelet levels of adrenaline (AD) and noradrenaline (NA). Patients were then allocated to groups according to the presence of OSA and/or hypertension.

**Results:**

A logistic regression model, controlled for age and BMI, showed that urinary AD and urinary NA were risk factors in the OSA+HYP group and the HYP group; however, the model showed higher levels of platelet NA for OSA without HYP. After 1 year of CPAP (continuous upper airway pressure) treatment, patients (n = 9) presented lower levels of urinary NA (p = 0.04) and platelet NA (p = 0.05).

**Conclusion:**

Urinary NA and AD levels were significantly associated with the condition of hypertension with and without OSA, whereas platelet NA with OSA without comorbidity. These findings suggest that platelet catecholamine levels might reflect nocturnal sympathetic activation in OSA patients without hypertension.

## Introduction

Measurement of catecholamine concentrations are requested often for the monitoring of many diseases, especially hypertension and pheochromocytoma [1], [2]. There is also a growing interest in obstructive sleep apnea (OSA) and its consequences [3]–[6]. Among these consequences, arterial hypertension (HYP) has been widely investigated [7]–[9]. In a recent update the authors reported the links between; hypertension, resistant hypertension and OSA [10].

Repetitive collapse of the upper airways during sleep, which may lead to arousal, hypoxemia, hypercapnia, and hemodynamic changes, contributes to increases in sympathetic tonus in OSA patients [11], [12]. The accumulation of sympathetic bouts during sleep following apnea events results in an increase in peripheral vascular resistance [13]. One of the most relevant candidates for the development of daytime HYP in OSA patients is chronic sympathetic activation [14]–[17]. Less attention has been paid to other mechanisms [18]–[21].

Plasma and urinary noradrenaline (NA) and adrenaline (AD) are frequently used in clinical settings to assess sympathetic status. However, these methods have limitations, which include unstable catecholamine levels, venous puncture influence, the effects of exercise and others [22]–[24]. In addition, their half-lives are short, which may preclude a more accurate estimation of their levels, particularly in OSA patients whose catecholamine levels may only be elevated during sleep [22], [23]. This may explain the controversy found in the literature when NA and AD levels are investigated in OSA patients [24]. In this context, Zweifler & Julius [25] argue that platelets constantly accumulate plasma-dependent catecholamine. While a plasma catecholamine levels fluctuate significantly, platelet catecholamine does not, and remain unchanged for 72 hours on average. This stability of catecholamine levels in platelets may facilitate its detection in patients with OSA, whose levels are mainly increased during sleep.

Coy *et al.*, [22] published a meta-analysis and observed similar difficulties due to an absence of standardized criteria for the assessment of monoamines. In addition, some studies have failed to control for confounding factors [22], [23]. Only 39% of studies controlled for dietary factors despite their documented influence on sympathetic nervous system (SNS) physiology. Increases in dietary sodium and carbohydrate levels have also been shown to affect catecholamine levels [20]. Obesity and hypertension are associated with high sympathetic tone and are also associated with OSA; both are major confounders when assessing sympathetic activity and catecholamine levels in OSA patients [7], [20], .

The objectives of this controlled study were to compare two different catecholamine laboratory tests, high pressure liquid chromatography (HPLC) and radioimmunoassay (RIA), and to validate and evaluate the platelet NA and AD assessment compared with urine and plasma tests in patients with OSA and hypertension.

The first experiment aimed to validate the methodology for measuring catecholamine levels in platelets and plasma of volunteers. Studies which assessed platelet catecholamine used HPLC technique. Most commercially RIA kits for adrenaline and noradrenaline are available to measure plasma and urine levels [26]. Therefore, we found important to validate platelet catecholamine dosage using both techniques RIA and HPLC comparing their performance with plasma, as a first step.

The second study compared OSA and HYP groups with regard to the NA and AD in urine, plasma and platelet tests. We also compared all catecholamine detection methods, in a subgroup of OSA patients, before and after CPAP (Continuous Positive Airway Pressure), the gold standard treatment for OSA [27].

We hypothesized that platelet catecholamine dosage is a better method to detect catecholamine elevation in OSA patients whose noradrenaline and adrenaline levels are mainly increased during sleep.

## Methods

### Experiment 1 – Validation

For this experiment 30 healthy, non-smoking volunteers (14 men), aged 18 to 55 years old (mean age 51.3±7.9) with mean BMI = 27.8±3.8 kg/m^2^ were evaluated. The volunteers were not under any medical treatment. Twenty ml of blood was obtained in EDTA with anticoagulant and plasmatic and platelet catecholamine levels were measured using both RIA and HPLC.

### Experiment 2 – Effect of OSA and HYP on catecholamine levels

A total of 156 subjects, from the Sleep Institute of Sao Paulo, Brazil, were consecutively enrolled in the protocol during the period from 2009 to 2011. They each underwent full polysomnography. Participants were included if they were aged between 30 and 65 years old (both genders), sedentary (exercise activity for at least 30 min on fewer than 3 days per week) and reported no recent hospitalization. The exclusion criteria were a body mass index (BMI) > 40 kg/m^2^, metabolic syndrome, pulmonary disease or New York Heart Association class III or IV heart failure, unstable angina, valvular heart disease, life-threatening arrhythmia, atrial fibrillation, left bundle branch block, uncontrolled hypertension, renal disease, use of any antiplatelet drug, pregnancy and those who were receiving treatment for OSA. All of these exclusion criteria were applied in a larger study of an OSA patient cohort [28]. An apnea-hypopnea index (AHI) ≥ 5 events/hour was considered to be diagnostic for OSA, and the control group was comprised of subjects with an AHI < 5 events/hour. The final sample was composed of four groups: Group I (n = 64, OSA and HYP), Group II (n = 50, OSA), Group III (n = 16, HYP) and Group IV (n = 24, Control). Eighty one percent of hypertensive patients used inhibitors of angiotensin converting enzyme (ACE) and/or hydrochlorothiazide, and 19% used beta-blockers. For ethical reasons, hypertensive patients already medicated were instructed to maintain their regular prescription, with the exception of an eventual dosage the night before blood collection. Patients were instructed not to take this nightly dose, but to wait and take it the next day only after the collection of biological materials.

The study was approved by the Ethics Committee of Federal University of Sao Paulo (CEP 0053; 10) and was in compliance with the Declaration of Helsinki II. All individuals signed the informed consent form, a copy of which was kept in their medical chart at Sleep Institute.

### Clinical evaluation

A staff cardiologist examined all patients and control subjects. The medical evaluation included blood pressure (for hypertension diagnosis, according to the seventh report of the Joint National Committee on Prevention, Detection, Evaluation and Treatment of High Blood Pressure and anthropometric parameters [29], weight and height measurements, and neck, waist and hip circumferences. Lung function testing was performed following the procedures, and the percentage of predicted values recommended by the American Thoracic Society was used to exclude pulmonary disease [30].

### Polysomnography

Overnight polysomnography was performed using the digital system EMBLA (17 channels, Natus Neurology, Ontario, Canada). The following variables were monitored: electroencephalogram (C3-A2, C4-A1, O1-A2, O2-A1), electroocculogram, electromyogram (submental and anterior tibialis muscles), electrocardiogram (ECG), presence of snoring, and body position. Chest and abdominal piezo-sensors monitored respiratory effort. Arterial oxygen saturation (SaO_2_) was recorded with a pulse oximeter. All polysomnographies were performed and scored by an experienced sleep technician and a sleep physician following the guidelines for sleep studies [31]. Arousals and respiratory events [30] were defined using the standard criteria [31]. A second full polysomnography was performed for CPAP (continuous upper airway pressure) titration in all OSA patients,

### Laboratory tests

The laboratory tests were performed in two steps. First, on the day of clinical evaluation, participants were asked to have a twelve hour fasting period and to come to the Sleep Clinic at 8:00 AM for blood sample collection for hemogram, creatinine, urea, glycemia, lipid profile, and hepatic function measures. The biochemical profile and the hepatic function measures were performed by an automated assay (ADVIA 1650, Siemens), and the hemogram was performed by microscopic and automated methods (ADVIA 120, Siemens).

One week later, participants returned to the Sleep Clinic for determination of urinary, plasma and platelet catecholamine. They were asked to abstain from a hypercaloric diet, alcohol, caffeine, chocolate, carbonated beverages, and tea three days before collection of blood and 24-hour urine [32]. Blood samples for plasma and platelet catecholamine measurements were obtained from an antecubital vein. The samples were collected in the morning at 9 AM, after a 12-hour fasting period and 1 hour of rest. Twenty four hour urine was collected from participants and then acidified with 5 mL of 6N HCl prior to the tests [33].

### Preparation of blood samples

The blood samples were immediately brought to the laboratory and prepared as described below:

Twenty milliliters of blood was collected in tubes containing 50 µL of 0.2 M NaEDTA solution per milliliter [34]. The platelet separation technical procedure followed the procedure described by Chamberlain *et al.*, [34] and cited by Christensen *et al.*, [35] The supernatants were decanted, and platelets were frozen and stored at −80°C until the analysis could be performed [34].

Release of catecholamine from platelet granules was assured by the addition of 10 µL 10% Triton X-100 to each 240 µL of platelet suspension after thawing. Otherwise, platelet suspensions were assayed in the same way as plasma samples, and the results are expressed relative to platelet number (pg/10^8^) [34].

Plasma: 2.5 mL of blood was collected in tubes, as previously described, and centrifuged for 10 min at 1800 g. The plasma was stored at −80°C until the analysis could be performed. All tests were performed in duplicate in each assay. Internal standards were used with every sample, and tests were analyzed according to reference values for each assay.

NA and AD concentrations in plasma, urine and platelets were quantified by a sensitive and accurate radioenzymatic assay with reagents from DIAsource Immunoassays S.A. (Nivelles, Belgium; Genese, Sao Paulo, Brazil). All tests were performed in duplicate.

The cutoff suggested by the manufacturer of the reagents is < 20 µg/24 hours for urinary adrenaline and < 100 pg/mL for plasmatic adrenaline; the cutoff is < 90 µg/24 hours for urinary noradrenaline and < 600 pg/mL for plasmatic noradrenaline. In the present study, we estimated cutoff levels for the different assessments, i.e., platelet, urine and plasma assessments for our population.

### OSA treatment

Of 114 patients who were offered CPAP, only nine patients successfully completed one year of CPAP use (compliance measure of smart card considered > 4 hours a day for at least 70% of the time) [36]. It is relevant to mention that there is no reimbursement for CPAP in most Brazilian states, which means that patients must pay for the treatment costs. These 9 patients were reevaluated after one year.

### Statistical analysis

In Study 1, Pearson correlations and coefficients of variation between and within assays (CV %) were calculated. For Study 2, descriptive analysis was based on the comparison of the four groups performed by ANCOVA with age, abdominal circumference and BMI as covariates. A Spearman correlation test was used to verify whether the three methods were comparable. We tested whether the standardized cutoff values of the tests were associated with clinical diagnoses of HYP and moderate or severe OSA (with or without HYP) using receiver operating characteristic (ROC) curves. Because cutoff values are only available for plasma and urine measurements, we used ROC curves to adjust the standard cutoff values for urine and plasma and to find new indicators in platelet analysis for the clinical diagnosis of HYP and OSA (with or without HYP).

The ROC curve was also used to obtain the sensitivity and specificity for different cutoff points. The sensitivity and specificity of urinary AD and NA (UAD and UNA), plasmatic AD and NA (PLAD and PLNA), and platelet AD and NA (PTAD and PTNA) measures for the detection of HYP and OSA, as well as their odds ratio and the calculation of the area under the curve (AUC) were analyzed. Finally, confidence intervals of catecholamine levels were calculated using the bootstrapping method to maximize the accuracy of measurement for each clinical condition, i.e., OSA and HYP.

Based on the new cutoff points found for each clinical condition, binary logistic regressions (controlled for BMI and age) were carried out to test this new measurement. The following independent variables were considered: biochemical measures of platelet in the different groups (OSA and HYP), and only OSA or HYP.

The sample size and Power analysis was calculated using GPower software version 3.1.2 (2009). With a priori expected effect size of 15% for differences among groups, observed Power approximately 80% and significance level 5% (p<0.05), our minimum sample size is 102 participants. By avoiding missing data and drop-outs during the study, we increased our data by 50%, yielding an actual sample of 154 participants. The analyses were conducted using “R” version 2.10.0 (R Development Core Team, 2009).

## Results

### Experiment 1

**Figure 1:**
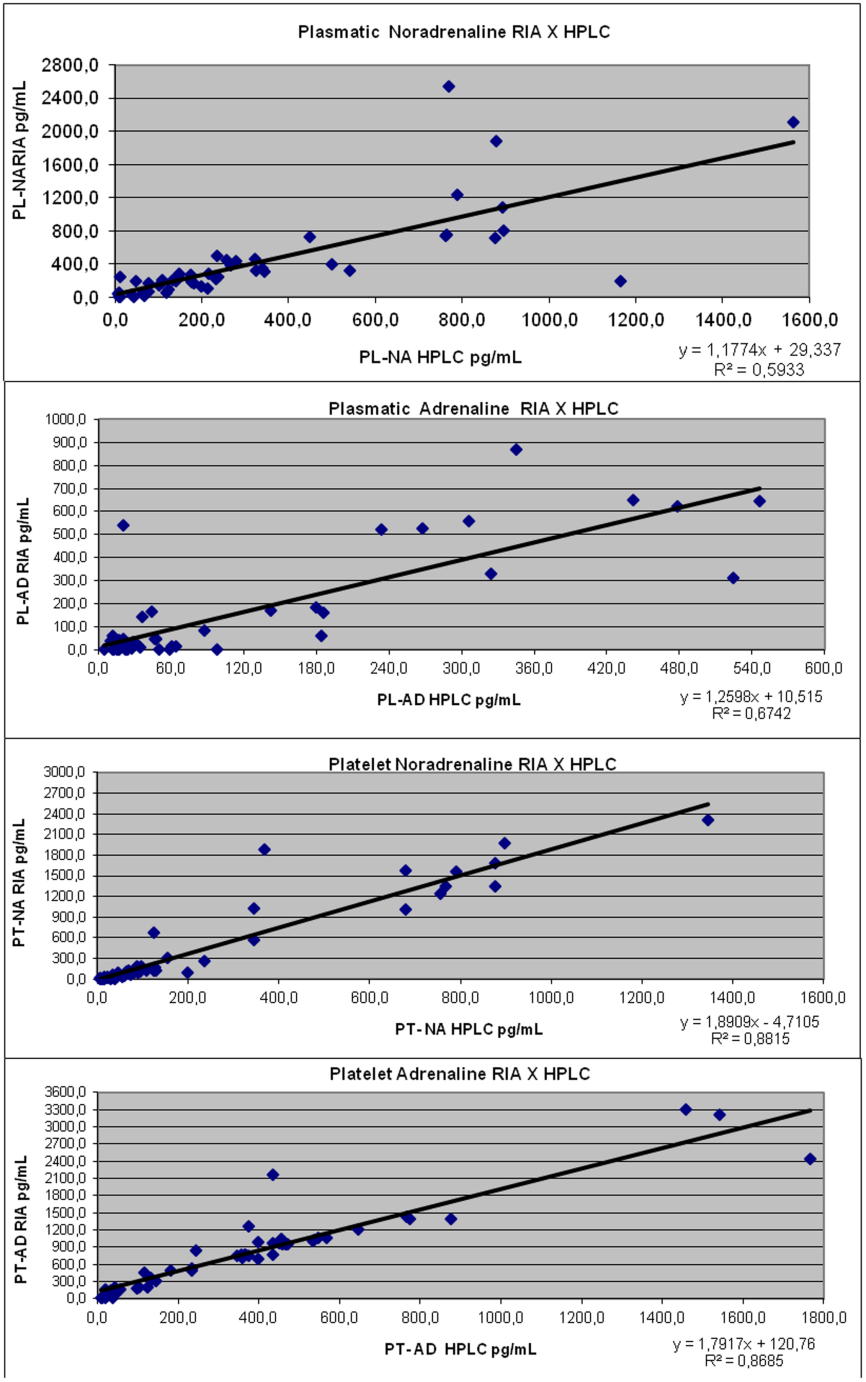
Correlation tests between methods of platelets and plasmatic catecholamine dosage RIA X HPLC.

The measurements of AD and NA in platelets by RIA and HPLC were compared. Figure 1 shows correlation results of both measures. Significant correlations were found between plasma RIA and HPLC for both AD and NA (r = 0.82 and r = 0.77, respectively). Similar results were obtained for AD and NA platelet assays, (r = 0.93 and r = 0.92). Due to good correlation results between NA and AD RIA and HPLC, RIA was chosen, based on the Westgard J. protocol [37] (**Figure 1**).

The coefficients of variation (CV) for AD and NA plasma were significantly higher than the platelet CVs. Adrenaline results: (plasma CV = 35±12.3% and platelet CV = 25±9.5%). Noradrenaline results: (plasma CV = 28±9.4% and platelet CV = 23±17.2%). In the light of these validation results we performed RIA in experiment 2.

### Experiment 2

The sample consisted of 156 individuals (91 women and 65 men) with a mean age of 50.8±7.9 years and an average BMI of 27.6±3.6 kg/m^2^.

We excluded two volunteers who did not complete the blood collection, as well as one volunteer who had exerted physical effort the day before blood sampling. The general characteristics of the groups' mean of anthropometric, polysomnographic and laboratory measures, including the catecholamine levels of the study sample, are shown in **Table 1** **.**

**Table 1:** Anthropometric, clinical, polysomnographic and catecholamine data, presented as means and standard deviations and corresponding to the baseline characteristics of the groups.

Number of cases (154)	Group I N = 64 OSA+HYP Mean±SD	Group II N = 50 OSA Mean±SD	Group III N = 16 HYP Mean±SD	Group IV N = 24 Control Mean±SD	P
**Age (Years)**	55.5±7.2	49.5±6.7	50.2±6.2	44.3±6.5	0.0007*
**Male gender (%)**	26	16	10	13	42%
**BMI (kg/m^2^)**	29.9±3.2	28.0±3.3	28.8±3.7	25.4±4.1	0.0008*
**Cervical circumference (cm)**	37.0±0.7	36.3±0.7	33.6±2.2	31.4±1.1	0.0009*
**Abdominal circumference (cm)**	94.4±1.4	95.1±1.4	91.6±4.4	92.6±2.3	0.0401*
**Hip circumference (cm)**	98.6±3.6	100.6±3.6	102.0±11.1	97.6±2.3	0.6500
**Systolic arterial pressure (mmHg)**	139.6±2.6	131.6±2.6	144.7±8.1	129.6±4.3	0.0006*
**Diastolic arterial pressure (mmHg)**	90.0±2.0	86.3±2.0	90.3±6.3	82.0±3.3	0.0006*
**AHI (events/h)**	21.3±3.2	22.2±3.2	3.2±3.9	5.4±5.2	0.0002*
**Total sleep time (min)**	340.6±65.1	332.0±106.1	267.9±184.3	349.6±99.6	0.2400
**Minimal saturation (%)**	85.6±1.4	83.9±1.5	89.0±4.5	89.4±2.4	0.0220*
**Arousal index**	18.4±3.0	23.1±3.1	7.3±9.4	14.8±4.9	0.0011*
**UAD (µg/24H)**	3.21±0.72	4.18±0.81	3.64±1.3	3.24±0.85	0.22
**PLAD (pg/mL)**	23.7±19.4	64.0±18.5	48.4±59.6	16.7±31.4	0.66
**PTAD (pg/10^8^platelets)**	128.3±86.1	115.6±86.4	148.4±254.0	111.4±139.1	0.89
**UNA (µg/24H)**	41.1±8.4	38.5±4.4	40.1±15.7	29.7±9.9	0.19
**PLNA (pg/mL)**	193.7±79.1	299.7±80.3	302.2±245.1	85.1±129.1	0.33
**PTNA (pg/10^8^platelets)**	651,1±506,7	557,7±451,7	511,6±528,6	381,2±209,5	0,10

^*^Significant value (p≤0.05).

We observed that the groups showed significant differences with respect to age (p <0.05): Group IV (control) was younger (44.3±6.5 years) than groups I, II and III (55.5±7.2, 49.5±6.7 and 50.2±6.2 years). BMI was also statistically significant (p = 0.0008) between groups I, II, and III (29.9±3.2, 28.0±3.3 and 28.8±3.7 kg/m^2^) when compared with the control group (25.4±4.1 kg/m^2^).

Table 1 depicts the average and standard deviations (adjusted for age and the BMI) of the anthropometric, polysomnographic, and laboratory catecholamine (AD and NA). Multiple comparisons of means were performed between groups.

The groups were significantly different when the anthropometric parameter variables were compared to those of the control group. Aside from age and BMI, neck circumference (p = 0.0009), abdominal circumference (p = 0.0401), systolic blood pressure (p = 0.0006) and diastolic blood pressure (p = 0.0006) all significantly differed among groups. Regarding polysomnographic data, significant differences were found in AHI (p = 0.0002), minimal saturation (p = 0.022) and arousal index (p = 0.001) as expected.

The results of biochemical tests for the study groups showed that urinary, plasma and platelet catecholamine levels were higher in OSA with HYP, OSA, and HYP groups when compared to the control groups; however, the results did not reach statistical significance **(** **Table 1** **)**. Similarly, the platelet catecholamine levels were also higher than the urinary and plasma levels in the same groups, but again did not reach statistical significance **(** **Table 1** **).**

### Biochemical test results

The correlations (**r**) in adrenaline measurements between methods ranged from 0.13 to 0.21, (weak and non-significant correlations), but for UAD vs. PTAD, the correlation was acceptable (r = 0.64, p = 0.02). The same was true for UNA vs. PTNA measures (r = 0.60, p = 0.01).

The following results refer to the analysis of the ROC curve for each analyte. We report the sensitivity and specificity for different cutoff points after comparison according to the HYP, OSA and OSA+HYP groups, as follows.

### Hypertension (HYP)

The new thresholds for UAD (≥ 4.21 µg/24H), UNA (≥ 40.0 µg/24H), PLAD (≥ 25.0 pg/mL), PLNA (≥ 170.0 pg/mL), PTAD (≥ 38.0 pg/10^8^), and PTNA (≥ 413.0 pg/10^8^) are displayed in Table 2. These results show that PLAD, PTAD and PTNA performed better for hypertension detection after using the new thresholds. Therefore, due to the actions of adrenaline/noradrenaline, patients who have values above the suggested bootstrap cutoff points are more prone to hypertension **(** **Table 2** **).**

**Table 2:** Results of the cutoff points obtained by the bootstrap for determination of AD and NA in different biological samples and groups.

Variables	Cutoff	present %	absence %	OR	p
***HYP group***					
**UAD**	≥ 4.21	9	5	1.8	p = 0.09
**(µg/24 h)**		64.2%,	35.8%		
**PLAD**	≥ 25.0	14	22	1.4	p = 0.03*
**(pg/ml)**		33.7%	56.3%		
**PTAD**	≥ 38.0	13	32	1.6	p = 0.01*
**(pg/10^8^platelets)**		28.8	71.2		
**UNA**	≥ 40.0	6	18	1.7	p = 0.05*
**(µg/24 h)**		56.0%	42.0%		
**PLNA**	≥ 170.0	9	15	1.4	p = 0.30
**(pg/ml)**		41.0%	59.0%		
**PTNA**	≥ 413.0	18	24	1.3	p = 0.03*
**(pg/10^8^platelets)**		52.0%	46.0%		
***OSA + HYP group***					
**UAD**	≥ 4.21	26	48	1.5	p = 0.00*
**(µg/24 h)**		55.0%	45.0%		
**PLAD**	≥ 25.0	24	18	1.3	p = 0.00*
**(pg/ml)**		57.0%	43.0%		
**PTAD**	≥ 38.0	49	18	2.7	p = 0.00*
**(pg/10^8^platelets)**		60.0%	40.0%		
**UNA**	≥ 40.0	25	17	1.4	p = 0.62
**(µg/24 h)**		40.0%	60.0%		
**PLNA**	≥ 170.0	32	45	1.3	p = 0.18
**(pg/ml)**		52.0%	48.0%		
**PTNA**	≥ 413.0	43	21	1.8	p = 0.01*
**(pg/10^8^platelets)**		57.0%	43.0%		
***OSA group***					
**UAD**	≥ 4.21	23	27	1.2	p = 0.65
**(µg/24 h)**		45.0%	55.0%		
**PLAD**	≥ 25.0	50	12	4.1	p = 0.01*
**(pg/ml)**		40.0%	60.0%		
**PTAD**	≥ 38.0	42	25	1.7	p = 0.02*
**(pg/10^8^platelets)**		32.0%	68.0%		
**UNA**	≥ 40.0	19	31	1.8	p = 0.03*
**(µg/24 h)**		42.0%	68.0%		
**PLNA**	≥ 170.0	32	18	1.7	p = 0.00*
**(pg/ml)**		48.0%	62.0%		
**PTNA**	≥ 413.0	28	45	1.4	p = 0.00*
**(pg/10^8^platelets)**		65.0%	35.0%		

^*^Significant value (p≤0.05).

A ROC curve was constructed for each platelet variable and the best hypertension predictors were found to be PTAD with 81% sensitivity and PTNA with 90% sensitivity, although the areas under the curve (AUC) were moderate, ranging from 67–81%. This was not sufficient to diagnose the HYP using the cutoffs from the literature **(** **Table 3** **).**

**Table 3:** Calculations of sensitivity, specificity and positive and negative predictive values of PTAD and PTNA.

Variables	Group I OSA + HYP	Group II OSA	Group III HYP	Group IV CONTROL
***PTAD (pg/10^8^platelets)***				
**sensitivity**	75%	67%	81%	79%
**specificity**	53%	73%	70%	61%
**value p+**	74%	78%	83%	62%
**value p-**	73%	57%	72%	80%
***PTNA (pg/10^8^platelets)***				
**sensitivity**	72%	85%	90%	74%
**specificity**	60%	73%	70%	75%
**value p+**	63%	85%	86%	85%
**value p-**	70%	76%	85%	74%

### OSA with HYP

The ROC curve showed that the best indicators for OSA with HYP, were UAD, PLAD, PTAD and PTNA levels (p = 0.00, p = 0.00, p = 0.00 and p = 0.01, respectively) **(** **Table 2** **)**.

The cutoff points, proposed by the bootstrap, and their sensitivities and specificities emphasize that PTNA (sensitivity = 72%) and PTAD (sensitivity = 75%) are not good indicators of interaction between OSA and HYP diagnoses **(** **Table 3** **)**.

### OSA

The ROC curve showed that PLAD, PTAD, UNA, PLNA and PTNA levels were significant indicators for detecting OSA alone (p = 0.01, p = 0.02, p = 0.03, p = 0.00 and p = 0.00, respectively). The sensitivity of diagnostic tests for OSA detection ranged from 67.0% for PLAD to 85.0% for PTNA (Table 2). Considering the cutoff points obtained by bootstrap indicators, PTAD showed 67% sensitivity; however, PTNA showed 85% sensitivity with 73% specificity for the diagnosis of OSA (Table 3).

### Logistic regression results

In Table 4, a binary logistic regression model was created where each measure was defined as a continuous independent variable, after which the capacity of continuous measurements to discriminate between each group (OSA+HYP, OSA and HYP) was verified. This was performed as a complementary analysis because it is a model that helps identify the factors that influence the risk of a clinical outcome, and it also enables the analysis of their respective odds ratios using newer reference cutoff points established through ROC curve analysis.

**Table 4:** Binary logistic regression for each clinical condition and their best markers (based on cutoff points found).

Group	Variables	Estimate	SE	LL95%	OR	UL95%	P
***HYP***	**UAD (µg/24 h)**	0.43	0.19	1.43	1.63	1.92	0.01*
	**UNA (µg/24 h)**	0.03	0.01	1.00	1.03	1.07	0.03*
***OSA***	**PTNA (pg/10^8^platelets)**	0.01	0.61	1.00	1.01	1.04	0.03*
***OSA+HYP***	**UAD (µg/24 h)**	0.59	0.22	1.35	1.55	1.85	0.00*
	**UNA (µg/24 h)**	0.04	0.02	1.00	1.01	1.09	0.01*

^*^ *Level of significance ≤ 0.05.*

The logistic model showed that an increase of 1 unit in the variable UAD resulted in a 1.63-fold increase in the chance of having HYP, while a 1-unit increase in UNA increased the chance of having HYP by 1.03-fold. Regarding OSA alone, a 1-unit increase of PTNA increased the chance of having OSA by 1.01-fold, whereas a 1-unit increase of UAD increased the chance of having OSA with HYP by 1.55-fold. Finally, a 1-unit increase in UNA increased the chance of having OSA and HYP by 1.01-fold.

Table 4 showed that UAD and UNA were the best predictors for HYP alone, with p = 0.01 and p = 0.03, respectively. Regarding OSA without HYP, only PTNA showed significance (p = 0.03) as a marker for identifying OSA. UAD and UNA were the best associated factors for OSA with HYP (p = 0.00 and p = 0.01, respectively).

### Group with one year of treatment with CPAP

Nine patients (3 female, 6 male) successfully completed one year of daily CPAP use. Their mean CPAP pressure was 10±3 cm H_2_O. Patients mean age was 56.0±5.2, mean AHI of 37.0±5.0, and they were all hypertensive.

When baseline values and those obtained after one-year of CPAP treatment were compared by a Wilcoxon test, we found significant decreases only for UNA (78.24±11.23 vs. 68.17±14.8; T = 9.0, Z = 1.59, p = 0.04) and PTNA (691.80±71.13 vs. 429.60±68.71; T = 7.0, Z = 1.83, p = 0.05) **(** **Table 5** **)**.

**Table 5:** Group with one year of treatment with CPAP.

Parameter	Initial average	Final average	Z	p
**UAD** µg/24 h	14,61	16,22	0,17	0,85
**UNA** µg/24 h	78,5	68,17	1,59	0.04*
**PLAD** pg/ml	26,94	77,22	1,48	0,13
**PLNA** pg/ml	134,8	198	0,53	0,59
**PTAD** pg/108 platelets	199	148,7	0,7	0,48
**PTNA** pg/108 platelets	691,8	429,6	1,83	0.05*

^*^ *Level of significance ≤ 0.05.*

## Discussion

Studying the catecholamine detection through laboratory measurements is a challenge due to confounding factors that influence the results of measuring urinary and plasmatic levels [38]. In addition, the physical and chemical properties of these neurotransmitters necessitate a careful choice of assessment methodology [39].

In the literature, there are several studies that assessed the SNS by determining catecholamine concentrations. Different techniques have been adopted, and the gold standard for catecholamine measurement is the HPLC method along with urinary measurement [26], [38], [39].

Our validation study for the platelet catecholamine method was to compare two methodological techniques, HPLC vs. RIA, which were not previously compared (experiment 1). The results showed a good correlation; therefore, we decided to use the RIA method. This method demonstrated the following advantages for this study: more acceptable percentages (as demonstrated by the coefficients of variation [CV%], intra and inter assay of platelet noradrenalin and adrenalin by RIA method), a greater ease and speed of execution, greater sensitivity and a smaller sample volume. In this way, our results substantiated our choice of this RIA method [40].

We did not perform validation methodology for 24-hour urine, because it is difficult to recruit volunteers to collect 24-hour urine. The authors focused on platelet and plasma catecholamine dosage, because platelet catecholamine concentration is dependent on the plasma pool. We acknowledge that muscle sympathetic activity, among all techniques, is the gold standard method to estimate sympathetic tone [41], [42]. However, is beyond the scope of this study to compare other techniques for the sympathetic activity assessment, other than body fluid analysis. We actually focused on catecholamine detection and laboratorial assays.

In experiment 2, analysis of the levels of catecholamine (AD and NA) in the three biological materials showed poor correlation between AD and NA levels in plasma and platelets; however, the study showed a strong correlation between urinary AD and NA levels.

This result is consistent with expectations because the urinary measurements reflect the overall rate of catecholamine excretion, and the catecholamine platelets, which are dependent on the plasma release pool, are able to retain catecholamine longer (48 to 72 hours); this is unlike plasma catecholamine, which reflects its very short half-life. The results of our analysis showed that the methods of measuring catecholamine levels assess catecholamine-release differently.

Measured concentrations of urinary catecholamine metabolites and their products can be used as markers of sympathetic tone during urine collection [38], [39], [41]. In children with OSA an overnight increase in urinary concentrations of catecholamine was found [42]. There are many problems inherent in the evaluation of plasma catecholamine levels, in particular as a SNS activation index, because these concentrations show large intra-variability that is exacerbated by factors such as venipuncture, stress, and physical exertion. These problems, which can result in increased variability, could be partially solved using the urinary catecholamine (urine, 24 h) measurement methodology. There are accuracy problems with this collection procedure, and studies that included analysis of intra-individual variability in catecholamine urinary concentrations suggest that the plasma measurements are higher [39], [40]. There are, however, studies in the literature that consider urinary catecholamine levels and their metabolites as the best parameters in OSA and HYP studies [41]–[44].

Mechanisms have been proposed to explain why OSA increases sympathetic tone. Usually sympathetic nerve activity, heart rate and blood pressure levels decline from wakefulness to non-REM sleep and increase again during REM sleep in normal sleepers without OSA. Stimulation of awakening is accompanied by increased sympathetic nerve activity, leading to transient increases in blood pressure [43]. It is likely that over time the OSA patient will continue to have increased sympathetic activity during the day, or at least in the early morning hours [41]. Thus, the significant association between HYP and OSA may be mediated by increased SNS activity and its effectors, noradrenaline and adrenaline, contributing to increased cardiovascular morbidity and mortality in these patients [6]–[10], [18].

A simple method that could detect such an increase in sympathetic activity in patients with OSA (treated or untreated) is of great value in clinical settings. This is the first study to evaluate platelet catecholamine content in OSA associated with hypertension comorbidity, a method widely used for pheochromocytoma diagnosis. This method has advantages related mainly to increased catecholamine availability in platelets, which ranges from several minutes in plasma (half-life) to 48 hours in platelets [43], [44]. There are also no alterations associated with sudden changes, such as stress, exercise and diet [19], [20], [18], [44]–[47].

The ROC curve analysis demonstrated low sensitivity among the methods (urine, plasma and platelet), in part because a subset (71%) of the total number of patients with hypertension (n = 54) was treated with medications that could potentially affect catecholamine levels. Although 81% of hypertensive patients did not use beta-blockers, they did use inhibitors of angiotensin converting enzyme (ACE) and/or hydrochlorothiazide. For ethical reasons, hypertensive patients who were already taking medications were instructed to maintain their regular prescription; however, they were instructed to refrain from taking antihypertensive medication the night before blood collection, and to only take it after the collection of biological materials.

Because platelet catecholamine had not yet been studied in these clinical populations, we used the bootstrap analysis to determine cutoff points for the proposed method, increasing the sensitivity and specificity of the method on the conditions under study, as presented in Tables 2 and 3. We found that the bootstrap conditions for sleep apnea and hypertension were, on average, 33% lower than the values found in the literature for the general population. It might be explained by the fact that our sample does not represent the general population, since it included patients who seek treatment for their conditions. However, we can assume that our data present high level of Interval Validity (with regard to Homogeneity of sample), which is relevant considering the objective of this study. Potential low External Validity can be associated with this lower cutoff because another variables found in our population may not be significantly present in the general population, such as high sympathetic activation, yielding lower cutoffs values to discriminate patients with OSA or Hypertension.

Urinary adrenaline and noradrenaline reference values were then considered to be ≤ 20 µg/24 h and ≤ 90 µg/24 h, respectively, and for plasma adrenaline and noradrenaline the values were ≤ 100 pg/mL and ≤ 600 pg/mL, respectively.

Finally, because the samples were consecutively selected at the Outpatient Sleep Institute, we were careful to statistically adjust the variables of age and BMI in the models. The standard diet for collection of biological material was respected and was one of the inclusion criteria.

The main findings of this study were the differential high levels of catecholamine in urine, platelets and plasma in the population with associated OSA and HYP which may explain the controversial results previously reported [22]. In the population with OSA without HYP, noradrenaline in platelets (PTNA) showed a better response. This finding demonstrates that platelets are more sensitive in subjects with OSA who have not developed hypertension, and who probably have increased sympathetic activity exclusively during sleep. In hypertensive individuals without OSA, urinary adrenaline (UAD) and noradrenaline levels (UNA) were more sensitive independent of medication, suggesting that such urinary measurements may be higher in patients with hypertension, with or without OSA. Only platelet noradrenaline, which is synthesized in the central nervous system (not platelet adrenaline, which is mainly synthesized at the adrenal medulla level), was found to perform better for OSA recognition. Finally, some studies suggest an association between OSA and platelet aggregates and volume [48]–[50]. On the other hand, catecholamine also increase platelet aggregates increasing cardiovascular risk [48]. Whether this association influences the catecholamine platelet detection in OSA patients is not clear.

Interestingly, in a relatively small sample of 9 patients, after one year of treatment with CPAP, only the urinary and platelet NA decreased significantly, while all plasma assays and AD levels did not show improvement with CPAP. Indeed, some studies showed that catecholamine levels were attenuated after treatment, and may worse after its withdrawal, suggesting that catecholamine levels may serve as a surrogate biomarker of OSA severity and may be used to monitor patients responses to treatment [51]–[53].

Potential limitations of this study were the use of an indirect assay method (RIA), the use of a radioisotope in this method, and the relatively small sample of OSA patients successfully treated. Even with a small sample size for blood and urinary assays, we were able to suggest that platelet as well as urine assays might be superior to plasma tests in this clinical population. Future studies with a larger sample of OSA patients and randomized trials with CPAP are worthwhile to validate platelet catecholamine assessment, particularly NA assessment, in this population, and perhaps in resistant hypertension patients. In the clinical context, utilization of catecholamine levels as a potential biomarker of OSA with respect to diagnoses and therapy monitoring deserves further validation [53].

In conclusion, we found that urinary noradrenaline and adrenaline levels were significantly associated with the condition of hypertension with and without OSA, whereas platelet noradrenaline was superior in detecting OSA without comorbidity. These findings suggest that in OSA, nocturnal sympathetic activation may be better detected by a technique that increases catecholamine availability, such as platelet dosage.
